# Beyond the Stereotype of Tolerance: Diversified Milieu and Contextual Difference

**DOI:** 10.3390/bs14020126

**Published:** 2024-02-09

**Authors:** Zhen Yue, Kai Zhao, Shunyu Zhu, Yifan Hu

**Affiliations:** 1 School of Foreign Studies, Xi’an Jiaotong University, Xi’an 710049, China; yuezhen@mail.xjtu.edu.cn; 2 School of Economics and Finance, Xi’an Jiaotong University, Xi’an 710061, China; zhushun@stu.xjtu.edu.cn (S.Z.); yifanhu@stu.xjtu.edu.cn (Y.H.)

**Keywords:** preference, tolerance, world values survey, milieu, creative workers

## Abstract

We explore whether there are value preferences of creative workers in addition to tolerance and how these value preferences vary among different occupation categories and countries. We use a dataset of 1968 and 1076 observations in China and the U.S., respectively, from the World Values Survey dataset (2017–2020, wave 7) (WVS 7), with a Structure Equation Modelling (SEM) and Multinomial Logit Model (MLM) at the micro level. The findings reveal that (1) the Chinese sample is more likely to have a balanced preference of tolerance towards migrants, religions, and homosexuality, while the American sample’s preference of tolerance is much more likely to be interpreted as accepting homosexuality only; (2) the American sample also shows preferences towards responsibility, technology, work style, and political actions, while a preference for happiness and political actions is identified in the Chinese sample; and (3) with a higher level of creativity, the difference regarding understanding of tolerance is more likely to be highlighted between China and the U.S. This study provides a quite unconventional perspective for understanding the composition of preferences and, to a certain extent, reconciles the inconsistency between the theoretical advocacy of building up a selected milieu and the reality of creative workers’ blended value mix.

## 1. Introduction

In contrast to the assumption of homogeneity in capital and labour within the framework of classical economic theory, modern human capital theory emphasizes the positive role of heterogeneous labour competencies and skills in sustainable economic development [1,2]. In the past decade, the research on human capital and its economic and social benefits has gradually changed from broadly defined human capital, i.e., using higher education-related indicators as proxies for specific occupational or labour characteristics. For example, Glaeser (2010) [3] argues that entrepreneurship, as a form of human capital, is fundamental to generating creativity and has a significant impact on the sustainable development of cities and regional economies. Human capital can also be defined, e.g., as information and communication skills, innovation skills, and economic capabilities [4], or management/marketing skills, technology application skills, and R&D skills [5] in some scenarios.

Among the extensive studies exploring the specific role of human capital in regional and urban economic development, the creative class thesis [6] is not a pioneer in developing a cultural economy. Landry and Bianchini (1995) [7], for example, emphasize the role of building up selected amenities, arguing that creativity has the notable features of mobility and preferential attachment. They also explain the causal relationship between urban quality of life, diversity, just-in-time experiences, and socio-economic development. However, Florida’s theory, using the gay/lesbian distribution or density to express and measure diversity, as well as its multiple definitions of creativity and further elaboration of the creative class and the mechanism of economic and social development, seems to be more disruptive and easier to be implemented [8]. It quickly became one of the hottest topics discussed internationally in the first decade of the 21st century. However, a large number of follow-up studies criticize the creative class thesis from various perspectives regarding the division of creative occupations [9], the definition of a tolerant milieu [10], and the possible gentrification [11], social inequality [12], and building up a tolerant milieu. This also prompts Florida and supporters to reinterpret the creative class thesis. Nowadays, the concept of the creative class is often used in combination with other theories of social and economic development, for instance, using creative workers as highly skilled working groups to explore community or workplace environments that are more likely to spark innovation [13,14,15].

Nevertheless, with the rise of the knowledge economy, neither Florida nor critics seem to disagree that a good milieu stimulates innovation; the core issue is whether a good milieu simply means a tolerant milieu and to determine its causal relevance to socio-economic developments. However, most of the present studies regarding the measure of a tolerant milieu based on macro and regional characteristics are difficult to reflect the preference of the creative class [16]. Synthetical indicators, such as the number of coffee shops and museums or the lesbian/gay index as Florida advocated, in fact, represent neither the cultural activities of individuals nor the true level of tolerance; they are only designed in favour of some particular scenarios, which are, however, disconnected from economic realities. It is also a farfetched match that only assumes the distribution of creative workers is an epiphenomenon of existing soft and hard environmental conditions in a city or region, and scholars pay too much attention to the discussion of tolerance while ignoring other values and spiritual needs that may exist among creative workers. Thus, while the concept of the creative economy or the creative class has been repeatedly discussed and evolved within the last two decades, this strand of literature has not yet thoroughly understood the milieu preferences of creative workers in a broader socio-economic context.

Therefore, the aim of this study is to develop a broader and much more comprehensive empirical framework to understand the relationship between creative job outcomes and preferences of creative workers, which is beyond the narrative scope of tolerance, as Florida suggests. More specifically, our research objectives are to seek clear answers to the following: (1) among other preferences, such as responsibility, political action, government power, work style, happiness, and technology, whether tolerance is the most important factor in association with occupation outcomes, and (2) whether there is any significant difference in value preference between Chinese and American creative workers at both the aggregate and sub-categorical levels.

Being enlightened by the concept of creative occupation defined by Florida [6], this study develops a comparable dataset for both the U.S. and China based on the 2017–2020 World Values Survey Wave 7 (WVS 7) [17]. The forthcoming World Values Survey Wave 8 (WVS 8) is planned for the period 2024–2026, which means that WVS 7 is, so far, the most recent dataset we can access. The reasons for comparing the case of China and the U.S. are twofold: On the one hand, empirical evidence towards validating the concept of preference and its associated causal relationship with socio-economic development is extremely insufficient in the non-American/European contexts. In particular, fewer studies have an opportunity to conduct a comparative analysis because, in many developing countries, the statistical calibre is quite inconsistent and there are limited statistics on occupational divisions at regional levels. For instance, the European Labour Force Survey provides the 3-digital occupational information by regions based on 0.1% of the total population in each EU country. However, in China, the Chinese Labour Force Survey only shows rough occupational information with limited observations, which makes a comparative discussion very difficult. In comparison, WVS, to the authors’ best knowledge, has the most harmonized dataset across different counties based on similar sampling and data generation approaches. On the other hand, from the theoretical perspective, the changing global economic/political landscape in the past decade has endowed the development of the creative economy with new definitions. With the rise of the power of the global south and when the global competition is more likely to be related to the creative potential of people and soft power, such a northern economic knowledge-based theory cannot be simply framed as global; it must go through a reinterpretation with the support of empirical evidence from various contexts. With this in mind, the comparison between China and the U.S. represents one of the major global economic and cultural conflicts and will shed more light on understanding how to achieve the goal of common prosperity and development and fundamentally revisiting the original theoretical framework of the creative class thesis, which is based almost entirely on North American experiences. 

The contributions of this study are threefold. First, we construct a comprehensive analytical framework that includes creative workers’ self-evaluation on the degree of occupational creativity and provides a novel perspective that explicitly reveals the diversified composition of preferences compared to present studies using indirect and synthetic indicators, such as the density of libraries and coffee breaks, as proxies to roughly represent the level of tolerance. Second, this study, to a great extent, incorporates many other dimensions of soft determinants that are mentioned by Florida’s critics and other economic/sociological studies into an analytical framework, in addition to a simple discussion of tolerance preferences only; this is an extension of the current definition of creative workers’ preferences. Third, existing studies discussing the role of preferences are mainly based on the context of single countries. We are, therefore, motivated to examine the association between diversified preferences and occupation outcomes using multi-country data from both transition and developed countries. 

## 2. Literature Review

### 2.1. The Core Concept of the Creative Class Thesis

The early version of the seminal creative class thesis proposed by Florida emphasizes the importance of creative talent aggregation to regional development [6]. As he claims, a newly emerged talent group has its own values, lifestyle, work style, and many other unique preferences compared to the old generation in the era of post-industrial society. As a result, Florida defines this particular rising force as the creative class, which can be further categorized as the super creative core with technological creativity, such as scientists, engineers, and university teachers, the creative professionals with economic and managerial creativity, such as managers and high-tech technicians, and bohemians, such as artists, journalists, and professional athletes [8]. The core assertion of the creative class thesis is the positive causality between building up a tolerant milieu and a sustainable economic growth. The creative class, as the nexus of all other creative elements within an innovation system, prefer to move, live, and work in places with such a halo, which in turn drives the entire society forward [18]. 

### 2.2. Unrevealed Mystery: A Flaw Theory or a Methodological Debate

Not surprisingly, Florida’s simple logic that tolerance promotes growth received much criticism in the U.S. in the first decade of the 21st century. Extensive studies completely denied Florida’s assertation, asserting that it lacks basic philosophical underpinning and contextuality [11]. For instance, Markusen points out that the creative class thesis essentially ignored the discussion regarding the social identity of the African-American population; thus, such a class may not be demographically representative [19]. Putnam states that a simple increase in ethnic diversity does not create the prosperity of a tolerant society in the short term [20]. In comparison, Donegan et al. directly compare the statistical power of Florida’s gay/lesbian variable with other traditional socio-economic variables [21]. The findings reveal that the tolerance variable Florida used is not significantly associated with growth indicators in the U.S. 

However, it appears that only the Florida-style concept of tolerance is disliked, not the general concept of tolerance. It is undeniable that places matter in the era of the knowledge economy and a tolerant, open environment is essential to igniting creativity, which in turn promotes socio-economic development [22]. Zhang et al. link the environment about the tolerance of using social media with employee creativity [23]. Recent studies, even though disagreeing with Florida’s causal relationship between tolerance and growth, still posit that exploring the effect of tolerance or openness is meaningful in explaining creative behaviours at multidimensional levels. For instance, Alfken et al. reveal that an open and tolerant region attracts artists in Germany [24]. Bereitschaft and Cammack assert that a tolerant community is more likely to accept creative workers’ behaviours and reduce negative effects brought by possible discrimination [25]. In non-American contexts, Rao and Dai, similarly to Florida’s theory, further propose the concept of socio-economic diversity, including ethnic diversity, cultural diversity, language diversity, marital status diversity and the density of the gay/lesbian population [26]. Tarasova et al. reveal residents from regions with varying degrees of ethnic diversity have differences in the degree of cultural values [27]. You and Bie find that the level of tolerance matters in retaining creative workers around 2000 in Shenzhen China, but economic incentives have played a dominant role since 2010 [28]. 

In summary, current research leads to mixed conclusions regarding how the concept of tolerance can be appropriately understood. It appears that the current research is mired in a methodological debate instead of focusing on the realistic demands of creative workers. Most studies, to a certain degree, attempt to reveal how one or a group of synthetic variables outperform others in explaining socio-economic development. This ignores the creative class thesis proposed by Florida and his followers or other strands of literature investigating the impact of soft power, in which the ways that creative workers demand a level of tolerance or how the presence of tolerance connects to creativity remains unclear. Moreover, given the challenge of data inconsistency, comparable research on tolerance is even more difficult in a non-American context, which leads to the fact that many studies, with similar aims and scopes substantially explore different scenarios. For instance, the gay/lesbian data are not available in Europe or most parts of the world; thus, the ratio of migrants [29], household registration [30], and the location of bohemians [31] are alternatively used as proxies to represent the concept of tolerance which, however, cannot precisely duplicate Florida’s theoretical framework and empirical results. 

### 2.3. Cultural Tastes or More: Beyond the Vision of Tolerance

Today, the world is facing many more issues, such as gentrification, inequality, and unemployment, compared to the early years of the 21st century when Florida’s creative class was identified. Even Florida has redesigned his recommendations and has begun to focus on the fall of the middle class and social segregation [32]. These newly emerged socio-economic, historical, and political contexts together have largely redefined the previous unified view of cities’ and regions’ development trajectory [33]. From this perspective, even though the creative class thesis has been fundamentally revisited, e.g., being considered as a theoretical puzzle that has been further integrated with other theories [34], the contextuality and complexity of creative workers’ values have not yet been thoroughly understood [16]. This is a broader socio-economic perspective that goes beyond the scope of focusing only on cultural tastes such as tolerance and openness [35]. Therefore, our research provides a first contribution in directly illustrating that creative workers’ behaviours, choices, and lifestyles are characterized by distinct values in different contexts, in addition to a cultural consumption of tolerance. We integrate the classic concept of shared values and socio-political priorities within Florida’s original theoretical framework and expect to determine in more detail whether values are only symbols of cultural production and consumption or are more broadly important for social division and socio-economic inequality. 

### 2.4. Values and Other Class Theories

As mentioned above, Florida’s advocacy regarding creative workers’ preferences only partially describes the operational mechanism of the global creative economy. In fact, the philosophical underpinning of the creative class thesis does not follow the Marxian model of class analysis, even though it repeatedly highlights that creative workers have collective values and actions, such as unconventional lifestyles. Therefore, it appears that how a creative worker’s position is unique and how a creative worker is conscious of her/himself remain unclear. Bourdieu’s class theory may provide us with a new perspective to understand how workers define them as a class [36]. Within a similar social space and having similar habits and tastes, a group of people eventually process similar natures and class habits, which in turn guide them to have a common practice and construct, enhancing such a class division. A typical example is the concept of independent-minded X people who devote themselves to art, writing and creative work [37]. Taking into account all these class theories, this study proposes a much broader theoretical framework that incorporates most of the mainstream value and preference variables subject to the data availability of WVS. According to Rokeach, a value is “an enduring belief that a specific mode of conduct or end-state of existence is personally or socially preferable” [38] (p. 5), thus it is an essential reflection of preferences and contains various aspects, such as morality, identities, fairness, and ideals, which can be either specific or abstract [39]. Therefore, in line with the current debate regarding social justice, inequality, and anti-consumerism [12] in the development of the so-called creative economy, and substantially focusing on specific groups of talents, this study generates the following value-based variables to represent the multidimensional features of a preference that is rarely mentioned by present empirical studies.

The gay/lesbian index was initially used by Florida and is the most controversial component of the creative class thesis [6]. As Florida explains, if a local community can accept homosexual people or behaviours, it is tolerant of every other consideration. Despite the criticism of the concept of tolerance, such an indicator is difficult to be duplicated outside the U.S. at the aggregate level. However, extensive studies, from the perspective of management, confirm that tolerance towards homosexuals (e.g., pro-gay behaviours) is indeed associated with productivity and creativity changes [40]. Therefore, these mixed conclusions require further exploration regarding how such a concept affects socio-economic development. Many European studies, given the issue of data constraints, use the ratio of foreign-born workers/immigrants or the density of coffee breaks and museums to represent the level of tolerance [29]. However, this study argues that tolerance towards immigrants and religions is a different concept and value reflection compared to tolerance towards homosexuality. It is, to a certain degree, consistent with Florida’s notion of openness. 

We also include happiness as an important indicator for predicting creative performance associated with job outcomes, as extensive studies show that happiness, as an expression of subjective wellbeing, such as satisfaction or income, is associated with health status, social relationships, work performance, and creativity [41]. However, previous studies cannot accurately reflect this aspect at an aggregate level. Responsibility is defined both individually and corporately as an important factor that links to the level and features of creativity. Florida provides only a blurry explanation of this. For the creative class, the cost of enjoying major benefits of socio-economic development is the responsibility to convert other work groups to be creative [42]. However, the psychology and management literature reveals the positive role of responsibility in more detail, such as Kim et al., who find that the level of corporate social responsibility (CSR) positively affects employees’ creativity within an organization [43]. Therefore, our analysis also includes this measure.

Creative workers should not only have a tolerant and open mind towards religious/cultural diversity but also a clear preference towards the development of modern technology [42]. We notice that extensive studies have shown that technology development and usage are essential in developing creativity; thus, workers must have different perceptions, knowledge, and attitudes towards technology associated with their creative levels and features. One of the most critical arguments about the polarized promotion of cultural consumption is that such a development model should never deviate from the core values of a society: “democratic values, social solidarity and the capacity for réjouissance” [12] (p. 573). Even though creative workers somewhat prefer individuality and self-expression and are reluctant to conform to traditional norms and institutional directives [42] (p. 56), they also prefer to live in a place where “collective action is easier as the bonds of familiarity and trust facilitate consensus and collaboration” [44] (p. 9), and “the political activism is strong” [45] (p. 49). Therefore, preferences for political action and government rights are included for reflecting these patterns. Finally, the preference for work style is related to Florida’s notion of lifestyle. Creative workers should have notably different work styles and lifestyles compared to other occupation groups or older generations. For instance, creative workers generally prefer an autonomous/flexible work style [42,46]. Such a work style is not an absolute reflection of workload, but creative workers dislike routinized and repeated schedules.

## 3. Variable Definition and Model Specification

### 3.1. Variable Generation Benchmarks

As shown in Table 1, we follow the basic logic of defining a group of occupations proposed by the creative class thesis and generally categorize the occupation information extracted from WVS 7 based on their creativity features, following the principle from basic to abstract. For instance, we assume university teachers, doctors, scientists, engineers, and artists, for example, (group 1) have scientific, technological, and artistic creativity, which is significantly different from those occupations with managerial and economic creativity, including high-level managers, bankers, government employees, and service staff (group 2), and those occupations with technical creativity, such as skilled and semi-skilled workers (group 3). The basic category includes routinised occupations, such as farm workers, drivers, and cleaners (group 4). It is worth mentioning that we do not strictly follow Florida’s taxonomy of creative occupation divisions. The occupation codes WVS 7 provides do not accurately match up with Florida’s definition, such as service staff—semi-skilled workers defined as a service class that does not belong to the creative class—and the accessible data do not allow us to further decompose these occupation groups. However, most occupations identified in WVS 7 are consistent with the definition of super creative cores and creative professionals; thus, we assume they are the most important leading forces in the development of the knowledge economy followed by semi-skilled and routinised labour. Here, we do not advocate the notion of elitism that one group of occupations is more creative than others, but we acknowledge that their positions in the process of generating creative ideas or converting innovation are indeed different. Therefore, it is very likely that values/preferences are aligned with occupations [35].

Regarding independent variables, the value variables mentioned in Section 2.4 are included, i.e., tolerance towards gay/lesbian, tolerance towards foreigners and immigrants, and preferences towards happiness, political actions, social responsibility, workstyle, government rights and technology. Demographic variables are also included, such as age, education level, gender, type of employment, etc. We mainly use latent variables abstracted from the SEM model based on answers from interviewees in each dimension to interpret values and preferences. Compared to previous studies using synthetic indicators, such as the number of coffee shops or museums, our estimation is much more direct and accurate. However, it has to be admitted that our model specification is difficult to include all preferences and values mentioned by previous studies, such as class consciousness, as WVS does not provide efficient information in these directions. This is likely to lead to the issue of omitted variables. We will use multiple models to check the robustness of these results.

Here, it is also worth mentioning that the scenario we developed approximates an efficient labour market where workers select jobs according to their competencies, experiences or cognitions towards a particular welfare, as WVS adopts the random sampling approach with weights to represent features of real target populations. According to hedonic wage theory [47], workers also care about the quality of the work environment, such as flexibility, enjoyment, and challenge, which in turn acquire knowledge of implicit/shadow prices towards these attributes. Similarly, creative workers enjoy multidimensional work and life environments and are more likely to define enjoying values and preferences during the production process as a crucial component (i.e., psychic wage) of the total expected wage. With a higher level of creativity involved in workplaces, such a portion is likely to be larger, which means that these creative workers would care less about compensating wage differentials. Therefore, our model specification, both SEM and econometric model, reflects such a pattern of hedonism. The higher the level of creativity attached to a labour group, the higher the level of explanatory power of those determinants proposed, such as the preferences towards tolerance, happiness or political actions in explaining job outcomes at an equilibrium market point. We assume that everyone has been aligned to an appropriate job according to a satisfactory hedonic and utilitarian division.

### 3.2. Analytical Model: SEM and Multinomial Logit Model

The above discussion leads to our novel model specification from two perspectives: first, an SEM model is developed that includes all forementioned value variables as latent variables, and the constructs at the upper level are tolerance towards gays/lesbians, tolerance towards immigrants/religions, happiness, responsibility, attitude towards technology, political action, government right and work style, which further affects one’s creative job outcome defined in Table 1. 

Next, following Comunian et al., a multinomial logit model regarding how creative occupations respond to their preference division is empirically developed [48]. At this stage, the key step is that the above SEM produces all core independent variables, which, however, cannot be directly and precisely measured by observed survey data, then our econometric model is used to reveal the causal relationship between creative job outcomes at the market and associated preferences.

(1) $$\mathrm{Pr}\left(Y\;=0|X\right)=\frac{1}{e^{x\beta^{1}}+e^{x\beta^{2}}+e^{x\beta^{3}}+1}\;\mathrm{Pr}\left(Y\;=1|X\right)=\frac{e^{x\beta^{1}}}{e^{x\beta^{1}}+e^{x\beta^{2}}+e^{x\beta^{3}}+1}\;\mathrm{Pr}\left(Y\;=2|X\right)=\frac{e^{x\beta^{2}}}{e^{x\beta^{1}}+e^{x\beta^{2}}+e^{x\beta^{3}}+1}\;\mathrm{Pr}\left(Y\;=3|X\right)=\frac{e^{x\beta^{3}}}{e^{x\beta^{1}}+e^{x\beta^{2}}+e^{x\beta^{3}}+1}$$

In Equation (1), outcomes 3, 2, 1, and 0 recorded in Y represent four different occupation outcomes. We do not provide a more disaggregated division of job outcomes as the sample size is not big enough to support the basic calculation requirement of a multinomial logit model.

Based on Equation (1), Equation (2) provides a risk-relative ratio (RRR) for a one-unit change in independent variables, which represents different demographic features and values. Here, values are measured by the PCA method, which are latent variables. Demographic features and dependent variables are observed variables.

(2) $$\frac{\mathrm{Pr}\left(Y\;=1|X\right)}{\mathrm{Pr}\left(Y\;=0|X\right)}={\;e}^{x\beta^{1}}\;\frac{\mathrm{Pr}\left(Y\;=2|X\right)}{\mathrm{Pr}\left(Y\;=0|X\right)}={\;e}^{x\beta^{2}}\;\frac{\mathrm{Pr}\left(Y\;=3|X\right)}{\mathrm{Pr}\left(Y\;=0|X\right)}={\;e}^{x\beta^{3}}$$

## 4. Results and Analysis

### 4.1. SEM Analysis: A Preliminary Understanding

#### 4.1.1. Pre-Tests

Based on the available data in WVS wave 7, our approach follows the rigorous process of SEM analysis. First, we carefully go through more than 200 variables to make sure that there are sufficient observations/qualified variables to support the theoretical model mentioned above. We obtained 1968 and 1076 observations in China and the U.S. categories, respectively. Second, for generating valid constructs, we repeatedly select related variables in each value dimension until the values of factor loading, AVE (Average Variance Extracted), and CR (Composite Reliability) are all generally greater than the threshold values (see Table 2 and Table 3). Finally, SEM analysis is conducted, and the robustness of the results is checked by Standardized Root Mean Square Residual (SRMR), Root Mean Square Error of Approximation (RMSEA), Comparative Fit Index (CFI), and Tucker-Lewis index (TLI) tests.

#### 4.1.2. Preferences of Creative Workers

**Figure 1:**
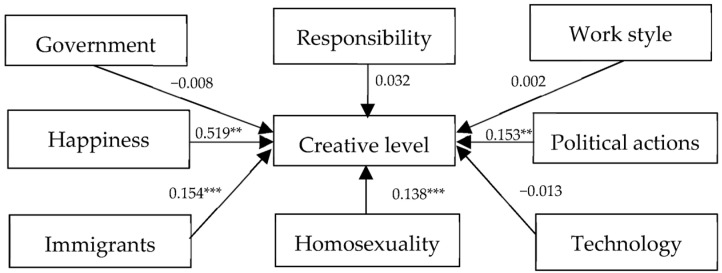
Results of the structural model analysis (China). SRMR = 0.063, RMSEA = 0.040, CFI = 0.914, and TLI = 0.902; a few values of AVE are below 0.5, but according to Fornell and Larcker (1981) [49], if the corresponding CRs are greater than 0.7, which is our case, the convergent validity of this construct is still adequate, ** *p* < 0.05, *** *p* < 0.01.

As shown in Figure 1, the results for the first time reveal a different occupational pattern of preferences in China compared to the case in the U.S. In the structural model, the latent variables for tolerance towards immigrants/religions, tolerance towards gays/lesbians, happiness, and political actions have significant effects on the level of creativity with positive standardized path coefficients of 0.154, 0.138, 0.519, and 0.153, respectively. 

**Figure 2:**
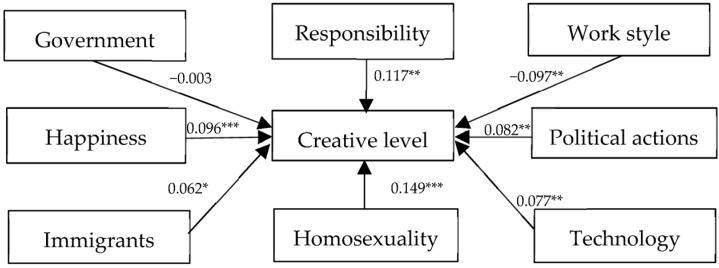
Results of the structural model analysis (U.S.). Notes: SRMR = 0.082, RMSEA = 0.042, CFI = 0.931, TLI = 0.911, * *p* < 0.10, ** *p* < 0.05, *** *p* < 0.01.

However, it must be admitted that the performance of the American sample, in some ways, verifies the core concept of the creative class at the micro level. As shown in Figure 2, the latent variables for tolerance towards immigrants/religions, gays/lesbians, technology, happiness, responsibility, work style, and political actions have significant positive effects on the level of creativity with positive standardized path coefficients of 0.062, 0.149, 0.077, 0.096, 0117, −0.097, and 0.082, respectively. 

### 4.2. Econometric Analysis: At a Further Disaggregate Level

The SEM model generally discusses which determinants would be associated with individuals’ creative levels. In comparison, our multinomial logit analysis further provides a detailed examination of how these effects vary at different creativity levels. In doing so, we initially save those valid contracts from our SEM analysis as independent variables, including happiness, responsibility, tolerance towards immigrants/religions and homosexuality, work style, government rights, and political actions; then a multinomial logit analysis is conducted with the control for demographic features of both samples.

Based on the approach of relative risk ratio (RRR), a ratio above one means that a relevant variable positively affects the outcome of obtaining a corresponding creative job compared to the base category (i.e., non-creative occupations). Table 4 and Table 5 present the results for China and the U.S., respectively.

In general, with the increase in creative levels, workers are more prone to have preferences, and this tendency is evident in both China and the U.S. For instance, choosing the preferences of happiness, political actions, tolerance towards immigrants/religion, and tolerance towards homosexuality increase the chance of having a job with scientific and artistic creativity by 149%, 653%, 463%, and 298%, respectively, in China. In comparison, with the preferences of responsibility and tolerance towards homosexuality, the chance of obtaining a job with scientific and artistic creativity increases by 170 times and 120 times, respectively, in the U.S. Regarding the preference of work style, Chinese and American scientific and artistic workers, however, exhibit a quite different feature. Even though such a preference is more likely to be associated with job outcomes with a higher level of creativity in both countries, a 41% decrease in probability of obtaining scientific and artistic jobs implies that American works do not agree with a conventional work style in this category (please see Table 2 and Table 3 for the definition of related questions), which is completely opposite to the case in China, where an approximately 60% increase is observed. As far as demographic features are concerned, it appears that education, age, and gender play the main roles in determining creative job outcomes. Particularly, workers are more likely to have a creative job with an age of about 20–31 in China and 32–46 in the U.S.

The independence of irrelevant alternatives (IIA) test indicates that using a multinomial logit model is feasible. However, the result based on a multinomial logit model is sensitive to sample size; thus, for testing the robustness of our results, the probit approach is employed to separately test whether the results are consistent between different sub-categories. We also further include the dummy variable that represents respondents’ parental occupations, as parents’ occupational choices are very likely to affect their children’s career trajectories [50]. Table 6 reveals results similar to those shown in Table 4 and Table 5. Overall, Chinese respondents are featured as more tolerant towards migrants and religions than towards homosexuality, compared to American respondents, who have a quite polarized tolerance preference towards homosexuality only. Moreover, it is interesting that parents’ occupational backgrounds have an impact on their children’s job outcomes in the sub-category with economic and managerial creativity, but for scientific and artistic jobs, or low-skilled jobs, respondents are less likely to be affected by parents.

Finally, this study also considers the possibility that work trajectories of creative workers could differ in another socio-economic dimension. For instance, preferences between poor and rich artists, or billionaire scientists and rookie scientists are very unlikely to be the same. With this in mind, we further use the variable of level of income (Q288R) to capture such a pattern. As shown in Table 7, probit analyses were only performed towards the sub-categories with the assumed highest level of creativity (i.e., specialists and technicians) and the second highest level of creativity (i.e., managerial and economic staff) in both the U.S. and China. Based on this specification, the baseline group in each sub-category includes those workers with a lower level of income. Here, we do not intend to discuss other sub-categories of creative labour, such as employees with technical or routinized jobs, because the effect of income is found to be minimal on job outcomes, i.e., the average income level is comparatively low for most of the employees within these categories and only a few observations can be defined as being wealth. We also attempt to compare the U.S. and China with other countries with a lower per capita income, such as Bangladesh, Ecuador or Myanmar, but similarly, only limited observations regarding high-level creativity jobs/incomes can be identified, which prevent us from conducting an accurate comparison. 

Overall, some similar differences and patterns as we captured in the aggregate analysis can be still found. Compared to the low-income group, a higher level of happiness and flexible workstyle and a lower level of tolerance towards immigrants/religion is more likely to be associated with richer specialists and technicians in the U.S. For managerial and economic staff, it appears that they dislike political actions. In China, only a higher level of happiness is associated with richer specialists and technicians, but interestingly, the lower level of tolerance towards immigrants/religion is found to be associated with the group of richer economics/managerial staff.

## 5. Discussions

Our SEM model specification is only employed for portraying a possible set of associations among different variables, but not causality, strictly speaking. These findings are still useful for understanding the nature of these creative workers in different socio-economic contexts. First, it appears that the underlying feature of occupational preference is not quite consistent with Florida’s proposition in China, as the dominant preference of tolerance is not identified, in association with job outcomes. With an increase in creativity level, happiness appears to be the most important factor in association with creative job outcomes in China and, surprisingly, Chinese creative workers are associated with a tolerant preference for both immigrants and gay/lesbian population or behaviours, and they are related to political actions to some extent. In comparison, the job outcomes of American creative workers are only more strongly associated with tolerance towards the gay/lesbian population, in addition to other multidimensional preferences, such as technology, happiness, responsibility, and work style. This can be deemed realistic as the WVS wave 7 data are mainly collected over the period 2018–2020. During the period, with Donald Trump’s presidency and his radical immigrant/foreign policy, the American people’s attitude towards such a sensitive issue is likely to be mixed. The result also implies that using immigrant-related indicators as a proxy for Florida’s gay/lesbian index is deceptively easy, but the results produced by this strand of literature are conceptually inconsistent. Additionally, we notice that both Chinese and American creative workers are prone to a positive political environment, but American creative workers are more willing to be political activists as a larger coefficient only implies a lower possibility, i.e., choosing the answer “would never do = 3”.

In summary, our SEM analysis reveals that there is a significant proportion of preferences attributable to the concept of tolerance, but it is defined differently by Chinese and American creative workers. However, it is also important to note that the composition of creative workers’ preferences is commonly blended; thus, only the total effect is associated with creative job outcomes.

Next, we use multinomial logit and probit models to further reveal the possible relationship between creative job outcomes and preferences. Compared to SEM, the econometric model somewhat further reveals a static causal relationship and facilitates sub-categorical analyses. Even though a few coefficients are no longer significant, such as technology and political action, the two approaches employed in this study, from quite different methodological perspectives, produce similar conclusions. First, with a higher level of creative job outcomes (i.e., in the occupational group of scientists), interviewees are more likely to be aligned to specific preferences, suggesting that a certain group of workers with different occupational backgrounds may indeed have a collective value mix or behaviours. This finding supports the general direction of developing creative labour/talents proposed by previous studies, such as investments in soft determinants in urban places. Second, Chinese creative workers are very less likely to participate in political actions as a large probability for category 3 implies that respondents are more likely to choose the answer “would never do = 3” relative to the base category. Finally, Chinese creative workers are much more likely to embrace the concept of tolerance towards immigrants/different religions, but American creative workers only prefer to accept the concept of tolerance towards homosexuality.

These findings, to a certain extent, confirm the advocacy of the hedonic wage theory [47]. Rich creative labour would care less about monetary incentives, thus demanding a higher level of psychic wage such as happiness, workstyle and tolerance. However, the quite different preference patterns are still evident between China and the U.S., similarly to the aggregate analysis, even though a new dimension, i.e., income level is incorporated.

It is worth mentioning that our model specification theoretically does not suffer a severe issue of endogeneity. The division of sub-categories is completely randomized based on the nature of the dataset; thus, a reverse causality does not exist, i.e., the ratio of one occupation group over another one has no impact on respondents’ value formation. An endogeneity caused by the presence of omitted variables is also less likely to affect the significance and sign of variables, as many conventional omitted factors, such as ability or experience that may affect income level, occupational choice, or human capital accumulation, do not have an effect on the probability of having different job outcomes in this context.

## 6. Conclusions and Implications

Based on SEM and logit/probit approaches, this study attempts to provide important and meaningful references for scholars and policymakers to understand the notion of preferences in a broader context. Even though quantitative datasets, such as WVS, do not provide lifetime tracking information towards interviewees before and after choosing specific jobs, which may not precisely simulate a real market environment and conclude a strict causality, our econometric analysis, incorporating the key variables produced by SEM model at the preliminary stage, still reveals several evident relationships between preferences and creative job outcomes. In the creative class thesis, Florida does not claim that other preferences of creative workers do not exist or are not important, but tolerance, as a dominant feature of the newborn class, appears to be a crucial catalyst to the long-run development of the knowledge economy in different socio-economic contexts. Therefore, if this advocacy can be proven, a creative worker should rank having a tolerant milieu as the most wanted preference. However, based on the data obtained from the latest edition of the World Values Survey (2017–2020), the findings, to a certain extent, show a great divergence in preferences among different creative occupation groups and in different contexts. It not only reflects the elements of tolerance and openness highlighted by Florida but also many more value dimensions fundamentally embedded in people’s daily lives [42]. At this point, our contribution is novel in providing a first detailed empirical analysis of values and preferences of creative occupations and it shows the distance between realistic values and attitudes of creative workers and the current research direction, which is, however, biased in demonstrating and representing preferences. It appears that some creative workers’ values are irrelevant to the culture produced or consumed, because universal values that are formed and developed during workers’ formative years are more influential or at least co-exist with the preference for tolerance and openness. Even in a situation where the component of tolerance is considered only within the theoretical framework of regional development, China’s case shows that the representation of tolerance is quite different compared to that of America.

Finally, regarding the theory of the creative class and others focusing on the production and consumption side of culture or a mixed approach [51,52,53,54], the ongoing conflicts in the geopolitical layout have already posed new challenges to policymakers and urban planners in the post-crisis era. The conundrum remains whether investing in hard policy measures outperforms soft ones in a specific context of regional development. If a local government is uncertain about which specific policy frameworks might be chosen, at least it should remain vigilant about the potential effects on the welfare of a society in the long run. Only highlighting the same preferences and values is insufficient to cope with the challenge of socio-economic inequality [55]. In many cases, policy interventions in institutional improvement, well-being, and social capital maybe more helpful than representation to satisfy creative workers’ total preference. Unlike America, with an evident preference towards a tolerance of homosexuality, as Florida suggested, investing in mixed and liberal values appears to be more feasible for developing countries such as China to develop a knowledge economy.

There are several limitations of this study. First, the cross-sectional nature of WVS data does not allow us to further capture time-varying features of observation. Second, WVS does not strictly control the data collection process exactly the same way in different countries; thus, our comparative analysis between China and America may have biases. Finally, it has to be admitted that the approach this study to define dependent variables, i.e., the level of creative jobs, is only an interpretation of a static market equilibrium, which cannot precisely capture dynamic patterns of a labour market, such as those who are left out of such jobs or those who are displaced from creative jobs and who, despite being creative workers, end up in less creative occupations or even in routine tasks. This is a common issue for questionnaire-based datasets, thus there is an urgent need for WVS to develop a more flexible dataset.

## Figures and Tables

**Table 1:** The definition of occupation outcomes and associated creativity levels.

	Occupational Groups	Importance to Knowledge Economy Development (CRE)	Observations	
			China	US
1	Specialists and technicians (e.g., university teachers, doctors, scientists, engineers)	Scientific, technological and artistic creativity, CRE = 3	284 (0.15)	482 (0.43)
2	Senior management (e.g., high-level managers, bankers, government employees)	Managerial and economic creativity, CRE = 2	778 (0.41)	428 (0.38)
3	Clerical staff (e.g., secretary, office manager, civil servant, bookkeeper)			
4	Sales personnel (e.g., sales managers, shopkeepers, salespeople, insurance agents, buyers)			
5	Service staff (e.g., restaurant owner, police officer, waiter, hairdresser)			
6	Factory technicians (e.g., foremen, motor mechanics, printers, tailors, mold makers, electricians)	Technical creativity, CRE = 1	451 (0.24)	153 (0.14)
7	Semi-skilled (e.g., bricklayer, bus driver, cannery worker, carpenter, sheet metal worker, baker)			
8	Non-technical personnel (e.g., porters, cleaners)	Routinised occupations, CRE = 0	373 (0.20)	64 (0.06)
9	Farm workers (e.g., farm workers, tractor drivers)			

**Table 2:** SEM pre-tests (China).

Variables	Description	Mean	Std	Factor Loadings	AVE	CR
Happiness	Construct					
Feeling of happiness	1–4 (very unhappy–very happy)	1.876	0.605	0.767	0.682	0.865
Satisfaction with your life	1–10 (dissatisfied–satisfied)	7.383	1.997	0.886		
Satisfaction with your financial situation	1–10 (dissatisfied–satisfied)	6.521	2.204	0.820		
Political actions	Construct					
Joining in boycotts	1–3 (have done/might do/would never do)	2.588	0.546	0.829	0.637	0.875
Attending lawful/peaceful demonstrations	1–3 (have done/might do/would never do)	2.663	0.496	0.847		
Joining unofficial strikes	1–3 (have done/might do/would never do)	2.704	0.503	0.764		
Signing a petition	1–3 (have done/might do/would never do)	2.408	0.621	0.749		
Attitude towards technology	Construct					
Because of science and technology, there will be more opportunities for the next	1–10 (completely disagree–completely agree)	8.624	1.682	0.845	0.667	0.857
Science and technology are making our lives healthier, easier, and more comfortable	1–10 (completely disagree–completely agree)	8.745	1.598	0.867		
The world is better off, or worse off, because of science and technology	1–10 (completely disagree–completely agree)	8.694	1.555	0.732		
Attitude towards government’s right	Construct					
Keep people under video surveillance in public areas	1–4 (definitely should not have the right (disagree)–definitely should have the right (agree))	1.819	0.888	0.748	0.667	0.857
Monitor all e-mails and any other information exchange	1–4 (definitely should not have the right (disagree)–definitely should have the right (agree))	2.303	0.998	0.862		
Collect information about anyone living in	1–4 (definitely should not have the right (disagree)–definitely should have the right (agree))	2.473	1.053	0.836		
Work style	Construct					
Work should always come first even if it means less spare time	1–5 (disagree strongly–agree strongly)	2.028	0.895	0.746	0.463	0.720
People who don’t work turn lazy	1–5 (disagree strongly–agree strongly)	1.942	1.008	0.631		
Work is a duty towards society	1–5 (disagree strongly–agree strongly)	2.103	0.909	0.658		
Responsibility	Construct					
National pride	1–4 (not at all proud–very proud)	1.665	0.643	0.711	0.478	0.731
Feel close to your country	1–4 (not close at all–very close)	1.759	0.655	0.761		
Willingness to fight for country	1–2 (no–yes)	1.104	0.306	0.592		
Tolerance towards different religions and immigrants	Construct					
People of a different race	1–2 (mentioned (disagree)–not mentioned (agree))	1.810	0.393	0.816	0.617	0.829
Immigrants/foreign workers	1–2 (mentioned (disagree)–not mentioned (agree)	1.751	0.432	0.787		
People of a different religion	1–2 (mentioned (disagree)–not mentioned (agree)	1.702	0.457	0.753		
Tolerance towards homosexuality	Construct					
Homosexuals	1–2 (mentioned–not mentioned)	1.283	0.451	0.778	0.570	0.798
Homosexual couples are as good parents as other couples	1–5 (disagree strongly–agree strongly)	3.672	0.939	0.789		
Justifiable: Homosexuality	1–10 (never justifiable–always justifiable)	2.288	2.424	0.694		

**Table 3:** SEM pre-tests (U.S.).

Variables	Description	Mean	Std	Factor Loadings	AVE	CR
Happiness	Construct					
Feeling of happiness	1–4 (very unhappy–very happy)	1.873	0.597	0.777	0.665	0.856
Satisfaction with your life	1–10 (dissatisfied–satisfied)	7.377	1.698	0.887		
Satisfaction with your financial situation	1–10 (dissatisfied–satisfied)	6.193	2.246	0.778		
Political actions	Construct					
Joining in boycotts	1–3 (have done/might do/would never do)	1.958	0.700	0.837	0.593	0.852
Attending lawful/peaceful demonstrations	1–3 (have done/might do/would never do)	1.985	0.667	0.828		
Joining unofficial strikes	1–3 (have done/might do/would never do)	2.280	0.589	0.742		
Signing a petition	1–3 (have done/might do/would never do)	1.418	0.605	0.659		
Attitude towards technology	Construct					
Because of science and technology, there will be more opportunities for the next	1–10 (completely disagree–completely agree)	7.279	2.262	0.857	0.712	0.881
Science and technology are making our lives healthier, easier, and more comfortable	1–10 (completely disagree–completely agree)	7.483	2.208	0.893		
The world is better off, or worse off, because of science and technology	1–10 (completely disagree–completely agree))	7.732	2.147	0.778		
Attitude towards government’s right	Construct					
Keep people under video surveillance in public areas	1–4 (definitely should not have the right (disagree)–definitely should have the right (agree))	2.200	0.880	0.642	0.616	0.826
Monitor all e-mails and any other information exchange	1–4 (definitely should not have the right (disagree)–definitely should have the right (agree))	3.183	0.857	0.860		
Collect information about anyone living in	1–4 (definitely should not have the right (disagree)–definitely should have the right (agree))	3.082	0.932	0.834		
Work style	Construct					
Work should always come first even if it means less spare time	1–5 (disagree strongly–agree strongly)	2.374	0.980	0.794	0.575	0.802
People who don’t work turn lazy	1–5 (disagree strongly–agree strongly)	2.629	1.088	0.783		
Work is a duty towards society	1–5 (disagree strongly–agree strongly)	3.320	1.093	0.694		
Responsibility	Construct					
Protecting environment vs. economic growth	1–2 (environment–economic growth)	1.409	0.492	0.629	0.501	0.749
Government’s vs individual’s responsibility	1–10 (government–people)	5.543	2.913	0.765		
Willingness to fight for country	1–2 (yes–no)	1.539	0.681	0.720		
Tolerance towards different religions and immigrants	Construct					
People of a different race	1–2 (mentioned (disagree)–not mentioned (agree))	1.973	0.161	0.715	0.535	0.774
Immigrants/foreign workers	1–2 (mentioned (disagree)–not mentioned (agree))	1.917	0.275	0.802		
People of a different religion	1–2 (mentioned (disagree)–not mentioned (agree))	1.978	0.147	0.671		
Tolerance towards homosexuality	Construct					
Homosexuals	1–2 (mentioned–not mentioned)	1.909	0.287	0.716	0.688	0.868
Homosexual couples are as good parents as other couples	1–5 (disagree strongly–agree strongly)	2.270	1.200	0.877		
Justifiable: Homosexuality	1–10 (never justifiable–always justifiable)	6.916	3.356	0.886		

**Table 4:** Multinomial logit analyses for Chinese creative workers.

	1 (Low Creativity)	2 (Medium Creativity)	3 (High Creativity)
Urban/Rural	3.779 *** (0.590)	4.927 *** (0.748)	6.513 *** (1.488)
Married/others	1.030 (0.245)	1.036 (0. 237)	0.896 (0.257)
Part time/full time	0.353 ** (0.120)	0.550 ** (0.167)	0.288 ** (0.141)
Self-employed	1.223 (0.212)	1.906 *** (0.315)	0.999 (0.240)
Male/female	2.685 *** (0.419)	1.004 (0.152)	1.050 (0.210)
Age 32–46/Age 20–31	0.819 (0.248)	0.969 (0.280)	0.840 (0.284)
Age 47–60	0.232 *** (0.066)	0.231 *** (0.063)	0.257 *** (0.087)
Age 60+	0.121 *** (0.038)	0.063 *** (0.020)	0.257 *** (0.087)
Bachelor degree and above/below	1.223 (0.586)	8.173 *** (3.562)	47.324 *** (21.302)
Happiness	1.157 (0.273)	1.238 (0.286)	2.489 ** (0.868)
Political action	1.156 (1.194)	3.058 ** (1.779)	7.527 ** (5.601)
Attitude towards technology	1.096 (0.075)	1.010 (0.065)	0.976 (0.086)
Attitude towards Government right	0.978 (0.170)	1.076 (0.181)	1.116 (0.252)
Work style	1.285 (0.292)	1.314 (0.285)	1.601 ** (0.470)
Responsibility	1.305 (0.482)	1.505 (0.536)	1.387 (0.659)
Tolerance towards immigrants/religion	2.116 ** (0.652)	3.974 *** (1.216)	5.629 *** (2.600)
Tolerance towards homosexuality	1.036 (0.440)	3.006 *** (0.748)	3.981 ** (1.898)
Constant	1.151 * (0.376)	3.582 *** (1.189)	0.275 *** (0.108)
Observation	1968		
Pseudo R^2^	0.22		

Notes: The variable definition behind the sign back slash is a base category and the same below; standard errors in parentheses * *p* < 0.10, ** *p* < 0.05, *** *p* < 0.01.

**Table 5:** Multinomial logit analyses for American creative workers.

	1 (Low Creativity)	2 (Medium Creativity)	3 (High Creativity)
Married/others	1.611 (0.562)	1.530 (0.495)	2.177 ** (0.726)
Part time/full time	0.641 (0.284)	0.631 (0.251)	0.491 * (0.205)
Self-employed	0.285 (0.165)	0.676 (0.321)	0.572 (0.281)
Male/female	1.486 * (0.564)	0.340 *** (0.136)	0.448 ** (0.157)
Age 32–46/Age 20–31	3.263 ** (1.692)	2.252 ** (1.063)	2.205* (1.059)
Age 47–60	1.704 (0.738)	0.766 (0.293)	0.749 (0.299)
Age 60+	1.698 (1.177)	0.849 (0.518)	0.771 (0.476)
Bachelor degree/below	1.080 (0.375)	3.171 *** (1.015)	18.837 *** (6.750)
Above bachelor degree	1.463 (1.750)	1.536 (1.781)	121.987 *** (130.52)
Happiness	0.647 (0.314)	0.897 (0.405)	1.444 (0.678)
Political action	0.017 (0.045)	0.029 (0.070)	0.427 (1.056)
Attitude towards technology	0.992 (0.098)	1.064 (0.099)	1.071 (0.103)
Attitude towards Government right	0.469 (0.386)	1.074 (0.815)	0.552 (0.434)
Work style	1.026 (0.317)	0.874 (0.248)	0.591 ** (0.172)
Responsibility	1.973 (3.169)	47.694 ** (69.672)	170.885 ** (254.690)
Tolerance towards immigrants/religion	0.091 (0.185)	0.259 (0.221)	2.2799 (4.933)
Tolerance towards homosexuality	3.090 (4.163)	50.071 *** (62.427)	121.089 *** (155.322)
Constant	1.285 (0.564)	10.177 *** (3.816)	2.985 *** (1.180)
Observation	1076		
Pseudo R^2^	0.19		

Note: standard errors in parentheses * *p* < 0.10, ** *p* < 0.05, *** *p* < 0.01.

**Table 6:** Analyses based on probit model at the sub-categorical level.

	1/0 (C)	1/0 (U)	2/0 (C)	2/0 (U)	3/0 (C)	3/0 (U)
Urban/Rural	0.713 *** (0.0996)	omitted	0.770 *** (0.0949)	omitted	0.910 *** (0.166)	omitted
Married/others	0.104 (0.151)	0.255 (0.213)	0.0300 (0.144)	0.146 (0.184)	0.0117 (0.233)	0.362 * (0.199)
Part time/full time	−0.462 ** (0.203)	−0.348 (0.276)	−0.490 ** (0.192)	−0.225 (0.226)	−0.801 ** (0.391)	−0.528 ** (0.256)
Self-employed	0.185^*^ (0.112)	−0.708* (0.372)	0.264 *** (0.102)	−0.174 (0.295)	−0.0144 (0.184)	−0.251 (0.301)
Male/female	0.535 *** (0.100)	0.272 (0.237)	−0.0137 (0.0957)	−0.578 *** (0.186)	0.0786 (0.161)	−0.563 *** (0.219)
Age 32–46/Age 20–31	0.00899 (0.186)	0.749 ** (0.318)	0.0742 (0.172)	0.442 * (0.245)	−0.0778 (0.262)	0.469 * (0.277)
Age 47–60	−0.695 *** (0.177)	0.388 (0.288)	−0.701 *** (0.164)	−0.166 (0.212)	−0.931 *** (0.253)	−0.193 (0.241)
Age 60+	−1.097 *** (0.196)	0.323 (0.445)	−1.362 *** (0.188)	−0.0378 (0.357)	−1.015 *** (0.292)	−0.133 (0.363)
Bachelor degree/below	−0.0847 (0.285)	−0.246 (0.237)	0.950 *** (0.214)	0.349 * (0.197)	1.978 *** (0.233)	1.161 *** (0.216)
Parents’ Job (L)/no job	−0.436 ** (0.178)	−4.817 (232.4)	−0.657 *** (0.165)	omitted	−0.660 ** (0.262)	−5.420 (192.2)
Parents’ Job (M)	0.207 (0.201)	−3.772 (232.4)	0.103 (0.183)	0.671 *** (0.221)	0.0841 (0.285)	−4.585 (192.2)
Parents’ Job (H)	−0.542 * (0.315)	−3.872 (232.4)	−0.276 (0.261)	0.729^**^ (0.310)	0.523 (0.353)	−4.066 (192.2)
Happiness	0.164 (0.153)	−0.178 (0.290)	0.0355 (0.143)	0.0423 (0.247)	0.424* (0.251)	0.0812 (0.278)
Political action	0.378 (0.382)	−2.304 (1.629)	0.643 * (0.359)	−1.959 (1.352)	0.394 (0.600)	−0.486 (1.478)
Attitude towards technology	0.0544 (0.0443)	0.0360 (0.0578)	−0.00927 (0.0414)	0.0530 (0.0528)	−0.0482 (0.0706)	0.0457 (0.0569)
Attitude towards Government right	0.0234 (0.110)	−0.563 (0.498)	0.112 (0.106)	0.0996 (0.417)	0.0866 (0.182)	−0.490 (0.472)
Work style	0.00383 (0.364)	−0.0266 (0.183)	0.284 (0.328)	0.0497 (0.163)	0.237 (0.575)	−0.178 (0.175)
Responsibility	0.0705 (0.227)	0.146 (1.045)	0.348 (0.222)	2.381 *** (0.820)	−0.0206 (0.366)	2.345 *** (0.867)
Tolerance towards immigrants/religion	0.333 * (0.198)	−1.725 (1.334)	0.855 *** (0.195)	−1.258 (1.242)	1.025 *** (0.372)	1.305 (1.450)
Tolerance towards homosexuality	0.122 (0.273)	1.137 (0.956)	0.751 *** (0.251)	2.300 *** (0.732)	0.706 * (0.409)	2.299 *** (0.743)
Constant	0.170 (0.265)	0.126 (0.283)	0.744 *** (0.249)	1.377 *** (0.200)	−0.387 (0.385)	0.750 *** (0.238)
Observation	887	201	1206	452	695	533
Pseudo R^2^	0.231	0.160	0.365	0.177	0.640	0.368

Notes: C = China/U = the U.S.; the definitions of job outcomes 0, 1, 2, 3 are as same as those in Table 1; standard errors in parentheses * *p* < 0.10, ** *p* < 0.05, *** *p* < 0.01.

**Table 7:** Analyses based on probit model towards particular sub-categories with different income levels.

	U.S.	U.S.	China	China
	Specialists and Technicians	Managerial and Economic Staff	Specialists and Technicians	Managerial and Economic Staff
Married/others	−0.0859	0.862 ***	−0.539 **	−0.223
	(0.204)	(0.275)	(0.275)	(0.275)
Part time/full time	0.00425	−0.460	−0.542	0.0338
	(0.309)	(0.371)	(0.476)	(0.473)
Self-employed	0.461	−0.354	−0.0747	0.383 *
	(0.284)	(0.547)	(0.283)	(0.203)
Male/female	0.639 ***	0.0231	0.147	0.311 *
	(0.219)	(0.315)	(0.184)	(0.181)
Age 32–46/Age 20–31	0.295	−0.190	−0.0647	0.172
	(0.256)	(0.393)	(0.239)	(0.255)
Age 47–60	0.690^**^	0.0535	−0.0592	−0.197
	(0.275)	(0.392)	(0.289)	(0.308)
Age 60+	0.864 **	0.822	−0.656	−0.0928
	(0.362)	(0.695)	(0.444)	(0.407)
Bachelor degree/below	0.660 *	0.350	−0.0399	0.264
	(0.358)	(0.323)	(0.232)	(0.214)
Parents’ Job (L)/no job	3.339	0.0896	−0.132	0.0744
	(264.6)	(1.001)	(0.364)	(0.333)
Parents’ Job (M)	3.473	0.839	−0.0336	0.0756
	(264.6)	(0.885)	(0.346)	(0.333)
Parents’ Job (H)	3.830	1.021	0.0516	0.114
	(264.6)	(0.937)	(0.376)	(0.473)
Happiness	1.045 ***	−0.410	1.391 ***	0.877 **
	(0.376)	(0.362)	(0.347)	(0.352)
Political action	−1.442	−5.146 **	0.151	0.706
	(1.520)	(2.173)	(0.664)	(0.620)
Attitude towards technology	−0.0247	0.00637	−0.0644	0.112
	(0.0652)	(0.0774)	(0.0843)	(0.0933)
Attitude towards Government left	0.170	−0.447	−0.289	−0.164
	(0.290)	(0.371)	(0.216)	(0.198)
Work style	0.292 *	0.279	0.145	0.0901
	(0.155)	(0.229)	(0.273)	(0.252)
Responsibility	−0.0693	−1.639	0.00897	0.0457
	(0.845)	(1.332)	(0.457)	(0.441)
Tolerance towards immigrants/religion	−2.544 *	−1.752	−0.114	−0.698 *
	(1.347)	(1.495)	(0.499)	(0.375)
Tolerance towards homosexuality	0.453	−1.508	0.640	0.348
	(0.822)	(1.226)	(0.407)	(0.388)
Constant	−6.385	−0.740	−0.740	−0.740
	(264.6)	(0.964)	(0.964)	(0.964)
Observation	478	146	285	796
Pseudo R^2^	0.19	0.10	0.11	0.11

Notes: standard errors in parentheses * *p* < 0.10, ** *p* < 0.05, *** *p* < 0.01; the reference group (0) is defined as those specialists and technicians or managerial and economic stuff with low-income status compared to their counterparts with high-income status (1).

## Data Availability

The data that support the findings of this study are available from the corresponding author, [Kai Zhao], upon reasonable request.
